# Anticoagulants beyond coagulation: repurposing heparin to mitigate immunothrombosis and inflammatory cascades in sepsis

**DOI:** 10.3389/fphar.2026.1901895

**Published:** 2026-10-06

**Authors:** Yanming Ren, Hongqi Meng, Beibei Li, Yuhao Wang, Lulu Gu, Ruyue Lv, Letian Xue, Li Xu, Hao Zhang, Dinghang Wang, Peng Sun

**Affiliations:** 1 Department of Emergency Medicine, Union Hospital, Tongji Medical College, Huazhong University of Science and Technology, Wuhan, China; 2 Key Laboratory of Anesthesiology and Resuscitation (Huazhong University of Science and Technology), Ministry of Education, Wuhan, China; 3 Hubei Provincial Engineering Research Center of Minimally Invasive Diagnosis and Treatment of Digestive System, Wuhan, China

**Keywords:** disseminated intravascular coagulation, endothelial glycocalyx, heparanase, heparin, histones, immunothrombosis, sepsis, sepsis-induced coagulopathy

## Abstract

Sepsis is a life-threatening syndrome caused by a dysregulated host response to infection, in which inflammation, coagulation, endothelial injury, and immune dysfunction interact to drive organ failure and death. Accumulating evidence identifies immunothrombosis as a central mechanism in this process, linking neutrophil extracellular traps, platelet activation, tissue factor expression, circulating histones, and glycocalyx degradation to microvascular collapse and multiple organ dysfunction. Although heparin has traditionally been used as an anticoagulant, its highly sulfated structure also permits interactions that are independent of antithrombin. In addition to inhibiting thrombin generation, heparin can neutralize extracellular histones, interfere with HMGB1- and lipopolysaccharide-related inflammatory signaling, inhibit heparanase, preserve endothelial glycocalyx integrity, and modulate leukocyte trafficking through selectin-related pathways. Most of these non-anticoagulant actions have been demonstrated in cell systems, animal models, human endotoxemia, or diseases other than sepsis, and should therefore be read as biological plausibility rather than as an established clinical effect. The clinical literature addresses three questions that should not be conflated: routine venous thromboembolism prophylaxis in critically ill patients, anticoagulation directed at sepsis-induced coagulopathy or disseminated intravascular coagulation, and experimental immunomodulatory repurposing. The most recent meta-analysis restricted to randomized trials found no significant mortality reduction with heparin across three trials and 426 patients (risk ratio 0.86, 95% confidence interval 0.60–1.24), and the disseminated intravascular coagulation subgroup was also inconclusive. Favorable associations reported in retrospective sepsis-induced coagulopathy cohorts justify prospective phenotype-enriched trials but do not identify established responders. This review summarizes the pathophysiological rationale, mechanistic basis, current clinical evidence, and major safety considerations of heparin use in sepsis, and highlights future directions including precision phenotyping, biomarker-guided therapy, and non-anticoagulant heparin derivatives. At present no heparin preparation, salt, dose, or biomarker threshold is validated for sepsis-specific immunomodulation.

## 1 Introduction

Sepsis is defined by the Sepsis-3 consensus as life-threatening organ dysfunction caused by a dysregulated host response to infection (Singer et al., 2016; Schlapbach et al., 2024). The global burden remains substantial; systematic analyses from the GBD 2021 study estimated 166 million cases and 21.4 million all-cause sepsis-related deaths in 2021, accounting for 31.5% of total global mortality (GBD, 2021 Global Sepsis Collaborators, 2025). This burden is particularly severe in children, with neonates and individuals younger than 19 years accounting for half of the approximately 50 million cases reported in the earlier GBD analysis (Rudd et al., 2020; Watson et al., 2024). The two figures are not directly comparable. The lower estimate comes from the GBD 2017 analysis, whereas the higher estimate comes from the GBD 2021 update, with differences in the observation period, input data, and estimation methods (Rudd et al., 2020; GBD, 2021 Global Sepsis Collaborators, 2025). The difference between these estimates should not be interpreted as a directly measured temporal increase in sepsis incidence. For children with suspected infection, the Phoenix Sepsis Score identifies sepsis at a score of at least 2, based on dysfunction in the respiratory, cardiovascular, coagulation, and neurological systems (Schlapbach et al., 2024).

Furthermore, complications such as sepsis-associated acute kidney injury (SA-AKI) are common in critically ill patients and are strongly associated with chronic morbidity and death (Zarbock et al., 2023). Immune dysfunction and susceptibility to ICU-acquired infections further complicate the course of critical illness (Kreitmann et al., 2024).

Historically, the clinical paradigm of sepsis-induced coagulopathy (SIC) focused on systemic, noxious activation of the coagulation cascade, which was viewed primarily as a deleterious precursor to disseminated intravascular coagulation (DIC) and multiorgan failure (Delabranche et al., 2017). Thrombin and fibrin generation were regarded as principal drivers of lethality, leading to large randomized trials that failed to improve survival through broad anticoagulation (Delabranche et al., 2017). Traditional diagnostic metrics also failed to capture the nuances of cellular hemostasis, including the coordinated activation of neutrophils, platelets, and endothelial cells (Delabranche et al., 2017). Contemporary thinking has shifted toward the concept of immunothrombosis, which reframes thrombosis as an intravascular effector of innate immunity (Engelmann and Massberg, 2013). This adaptive process coordinates blood coagulation and platelet activation to generate a physical scaffold that facilitates pathogen recognition, containment, and destruction (Engelmann and Massberg, 2013). Within this framework, neutrophils are central players, mediating procoagulant activity through the release of neutrophil extracellular traps (NETs)—complexes of cell-free DNA, histones, and granule proteins (Denning et al., 2019; Gould et al., 2015). Although NETs contribute to host defense by trapping microorganisms (Denning et al., 2019), their uncontrolled release triggers a dysregulated thromboinflammatory response that damages the microcirculation (Alsabani et al., 2022; Aklilu et al., 2025). In parallel, interactions among activated platelets, complement, and the endothelium promote pathological vascular occlusion and organ ischemia (Aklilu et al., 2025). Accordingly, sepsis is now increasingly understood as a transition from protective immunothrombosis to detrimental thromboinflammation (Retter et al., 2025). Defining this threshold is critical for precision medicine, as it shifts therapeutic focus from universal anticoagulation toward targeted interventions that preserve adaptive host responses while suppressing excessive thrombin generation (Delabranche et al., 2017; Aklilu et al., 2025).

Heparin, a mammalian polysaccharide, has traditionally been used as a prophylactic anticoagulant for thrombotic disorders (Derhaschnig et al., 2003). Pharmaceutical heparin is a linear, highly sulfated glycosaminoglycan extracted from porcine intestinal mucosa or, less commonly, from bovine lung or intestine. It consists of repeating uronic acid and glucosamine disaccharides carrying N- and O-sulfate groups, which give the molecule the highest negative charge density of any known biological macromolecule (Mulloy et al., 2016; Hogwood et al., 2023b). Unfractionated heparin (UFH) is a polydisperse mixture of chains of approximately 5–30 kDa; low-molecular-weight heparins (LMWH) are produced by controlled chemical or enzymatic depolymerization to a mean mass of about 4–6 kDa; and fondaparinux is a fully synthetic pentasaccharide (Mulloy et al., 2016; Hogwood et al., 2023b). Because chain length and sulfation determine which binding partners are accessible, the terminology in this review is deliberately specific: statements refer to UFH, to a named LMWH, to fondaparinux, or to a modified non-anticoagulant derivative rather than to heparin as a single agent. The counterion differs between products as well. Sodium and calcium heparin are both used therapeutically and are regarded as pharmacodynamically equivalent, whereas lithium heparin is a laboratory anticoagulant used in blood-collection tubes and is not administered to patients; no evidence indicates that the choice of salt alters sepsis outcomes (Mulloy et al., 2016; Hogwood et al., 2023b). Although the anticoagulant effects of heparin are well established, its proposed systemic anti-inflammatory effects have not been validated as a sepsis-specific clinical indication (Li et al., 2021). Evidence from murine experiments indicates that heparin prevents caspase-11-dependent septic lethality and heparanase-mediated macrophage glycocalyx degradation through mechanisms independent of its anticoagulant properties (Tang et al., 2021). Heparin also functions as an anti-histone agent by binding circulating histones, which act as damage-associated molecular patterns (DAMPs) that amplify inflammation and endothelial injury (Iba et al., 2015; Longstaff et al., 2016). Retrospective cohorts report higher survival among septic patients exposed to heparin, with matched hazard ratios of approximately 0.7–0.8 for early prophylactic exposure and larger apparent effects in coagulopathic subgroups; these are associations derived from observational data rather than randomized estimates of treatment effect (Zou et al., 2022; Peng et al., 2022). In human endotoxemia, UFH attenuated the lipopolysaccharide-induced fall in surface L-selectin on circulating leukocytes (Derhaschnig et al., 2003). Biochemical and experimental studies support competitive inhibition of L- and P-selectin ligand binding by heparin oligosaccharides, but do not establish why leukocyte surface L-selectin was preserved in the human endotoxemia study (Wang et al., 2002). Clinical use nonetheless remains largely confined to conventional anticoagulation rather than targeted immunomodulation.

Several reviews, including Li and Ma (2017) and Yu et al. (2024), have already catalogued the immunomodulatory actions of heparin. The question addressed here is narrower and more translational: do these non-anticoagulant actions justify any sepsis-specific use beyond routine venous thromboembolism (VTE) prophylaxis, and how should therapeutic intent, formulation, phenotype, evidence level, and safety alter that judgment? This review therefore differs from those earlier syntheses in four practical ways. First, claims are distinguished by evidence source, from established pharmacology through biochemical, cellular, animal, human endotoxemia, randomized, and observational evidence. Second, routine VTE prophylaxis, coagulopathy-directed anticoagulation, and experimental immunomodulation are treated as distinct clinical purposes rather than as one indication. Third, the divergence between null randomized results and favorable observational associations is examined directly, including confounding by contraindication and time-related exposure bias. Fourth, the identified gaps are translated into a trial framework that prespecifies phenotype, preparation, dose, target-engagement biomarkers, comparator, and safety outcomes. Although heparin is a century-old first-line anticoagulant, its structural properties enable selective interactions with multiple proteins, conferring anti-inflammatory, antiviral, and antitumor activities (Wang et al., 2022a; Song et al., 2024). Within that frame we examine the mechanistic rationale for repurposing heparin to treat sepsis-induced organ dysfunction, focusing on its ability to stabilize the vascular endothelial glycocalyx and to inhibit the toxic effects of DAMPs such as circulating histones and high-mobility group box 1 (HMGB1) (Li et al., 2021; Song et al., 2024; Uchimido et al., 2019). We also synthesize the clinical evidence, including machine-learning-based phenotyping and retrospective database analyses, in order to clarify what such studies can and cannot establish about early heparin exposure in patients with sepsis-induced coagulopathy (SIC) and multiple organ dysfunction syndrome (MODS) (Zou et al., 2022; Guo et al., 2022). Table 1 summarizes the principal clinical studies on common fields, Table 2 sets out the candidate enrichment phenotypes, and Figures 1, 2 map the molecular targets and the corresponding evidence level.

**TABLE 1:** Clinical studies and evidence syntheses of heparin exposure in sepsis or sepsis-associated coagulopathy.

Study	Design/n	Population	Preparation	Purpose and regimen	Timing/duration	Control	Mortality result	Bleeding/safety	Key limitation
Jaimes et al., 2009 (HETRASE)	Double-blind RCT; 319	ED patients with sepsis; no SIC/DIC enrichment	UFH	Treatment; 500 U/h continuous infusion	7 days	Placebo	28-day mortality 14% vs. 16%; p = 0.652; primary outcomes null	One serious UFH adverse event, resolved	Single center; fixed regimen; no phenotype enrichment
Levy et al., 2009 (XPRESS)	Double-blind RCT safety analysis; 1,994	Severe sepsis receiving DrotAA	UFH or enoxaparin	VTE prophylaxis; UFH 5,000 U q12 h or enoxaparin 40 mg qd	During 96-h DrotAA infusion; bleeding assessed days 0–6	Placebo plus DrotAA	No statistically significant mortality difference	Any bleeding 10.8% vs. 8.1%; p = 0.049; serious bleeding 2.3% vs. 2.5%; p = 0.72	Historical DrotAA context; prophylaxis, not immunomodulatory treatment
Liu et al., 2014	Prospective randomized study; 37	Sepsis with early DIC/pre-DIC	UFH	Treatment; 70 U/kg/day continuous infusion	5–7 days	Saline plus sepsis care	28-day mortality 31.8% vs. 40.0%; p = 0.434	Bleeding rate not clearly reported	Very small, single center; mortality underpowered
Weng et al., 2021	Prospective randomized 3-group study; 112 total; relevant n = 72	Sepsis-associated DIC; heparin and SHAM groups n = 36 each	Heparin sodium	Treatment; 50 mg/day by continuous 24-h pump	Daily treatment; outcomes through day 7 and day 28	Conventional treatment (SHAM group)	28-day mortality 33.3% vs. 38.9%	Bleeding events 20.0% vs. 44.4%	Heparin was a comparator in a plasma-exchange study; high risk for intervention deviations
Chen et al., 2023	Double-blind RCT; 95 (47 vs. 48)	Sepsis-associated acute gastrointestinal injury	LMWH sodium	Biomarker/organ-function treatment; 4,000 U SC qd	7 days	Saline SC plus conventional treatment	No significant difference in 28-day survival	No associated bleeding reported; event count not provided	Single center; biomarker-focused; not powered for survival
Totoki et al., 2024	Systematic review of 3 RCTs; n = 426	Adult sepsis; DIC subgroup n = 109	UFH/heparin sodium; regimens varied	Sepsis-directed treatment; 12,000 IU/day, 70 U/kg/day, or 50 mg/day	Varied	Placebo, saline plus sepsis care, or conventional treatment	Overall RR 0.86 (0.60–1.24); DIC RR 0.84 (0.51–1.38)	Bleeding RR 0.49 (0.24–0.99)	Only 3 small trials; heterogeneous designs and risk-of-bias concerns
Wang et al., 2014	Meta-analysis; 9 reports, 3,482	Sepsis/severe sepsis	UFH or LMWH	Mostly prophylactic or concomitant exposure; heterogeneous doses	Varied	Placebo/trial control	28-day mortality OR 0.656 (0.562–0.765)	Bleeding: 7 RCTs, n = 2,726; OR 1.063 (0.834–1.355)	Included exposure embedded in trials of other anticoagulants; low-quality small trials
Zarychanski et al., 2015	Systematic review; 9 RCTs, 2,637	Sepsis, shock, or infection-associated DIC	UFH or LMWH	Prophylactic or treatment exposure; varied	Varied	Placebo/usual care or other anticoagulant	Vs. placebo/usual care RR 0.88 (0.77–1.00)	Major bleeding RR 0.79 (0.53–1.17); sparse reporting	Mostly unclear risk of bias; treatment purpose heterogeneous
Zou et al., 2022	MIMIC-IV retrospective; 6,646; matched n = 5,416	Sepsis-3; SIC in 83.7%	UFH	VTE prophylaxis; 5,000 U SC per recorded dose	Started within 48 h	No early heparin	Matched in-hospital HR 0.78 (0.68–0.89)	Bleeding and HIT not assessed	Confounding by eligibility, procedures, and treatment trajectory
Peng et al., 2022	MIMIC-III retrospective; 1,820; 652 pairs	SIC score ≥4	UFH	Mixed prophylactic/treatment exposure; dose not standardized	During ICU stay; start not standardized	No UFH	Matched 28-day HR 0.323 (0.258–0.406)	Intracranial OR 1.48 (0.955–2.294); GI OR 1.094 (0.503–2.382)	Time-varying confounding; indication and dose heterogeneous
Huang et al., 2023	MIMIC-IV retrospective; 3,377; 784 pairs	SIC; subgroup SIC score 4	UFH	SC exposure; 6,250–13,750 IU/day signal	Within 24 h	No early UFH	Matched ICU HR 0.69 (0.52–0.92); SIC-4 28-day HR 1.08 (0.74–1.58)	Not reported	Observational dose-response; outcome inconsistency; residual confounding
Cai et al., 2023	MIMIC-IV latent class analysis; 4,993	SIC; 3 data-derived classes	Heparin not specified	Database exposure; dose and indication not standardized	Not standardized	No heparin within class	Class 2 adjusted 28-day OR 0.39 (0.30–0.49); no benefit in other classes	Not reported	Exploratory subgroup derived and tested in same database
Zhang Z et al., 2023	Single-center retrospective; 220 eligible; 88 pairs	Sepsis-3; SIC/DIC subgroups	LMWH, product unspecified	SC treatment; dose not reported	During admission; start not standardized	No LMWH	28-day mortality 26.1% vs. 42.0%; adjusted HR 0.50 (0.29–0.86)	Major bleeding 6.8% vs. 8.0%; p = 0.773	Small retrospective cohort; exposure details incomplete

Effect estimates are reported with 95% confidence intervals (CI) as given in the original reports. AGI, acute gastrointestinal injury; CI, confidence interval; DIC, disseminated intravascular coagulation; DrotAA, drotrecogin alfa (activated); ED, emergency department; GI, gastrointestinal; HIT, heparin-induced thrombocytopenia; HR, hazard ratio; ICU, intensive care unit; LMWH, low-molecular-weight heparin; MIMIC, Medical Information Mart for Intensive Care; OR, odds ratio; RCT, randomized controlled trial; RR, risk ratio; SC, subcutaneous; SIC, sepsis-induced coagulopathy; UFH, unfractionated heparin; VTE, venous thromboembolism.

**TABLE 2:** Clinically and molecularly defined sepsis phenotypes relevant to heparin trial design.

Phenotype	Definition/derivation	Characteristic features	Pragmatic indicators	Potential relevance to heparin	Evidence status	Important limitation
Sepsis-induced coagulopathy (SIC)	ISTH SIC score ≥4; combined platelet/PT-INR score >2 (Iba et al., 2017)	Early platelet decline, PT-INR prolongation, organ dysfunction	Platelets, PT-INR, four-organ SOFA	Enrichment for coagulation-active sepsis	Consensus score; retrospective heparin associations	Not a validated heparin-response rule; bleeding phenotype may coexist
ISTH overt DIC	Score ≥5 using platelets, fibrin markers, PT and fibrinogen (Iba et al., 2023b)	Consumptive coagulopathy, high fibrin turnover, thrombosis and/or bleeding	Platelets, D-dimer/FDP, PT, fibrinogen	Identifies advanced coagulation dysregulation	Established DIC classification; randomized heparin subgroup inconclusive	Late and heterogeneous; not all DIC is thrombotic-dominant
SIC latent Class 2	Unsupervised class in MIMIC-IV (Cai et al., 2023)	High PT/INR, lactate, SOFA and mortality; multiple-organ dysfunction	Routine clinical and coagulation variables	Observed association with lower mortality during heparin exposure	Exploratory retrospective signal	Class derived and evaluated in same database; dose, timing and safety incomplete
Clinical alpha/beta/gamma/delta phenotypes	Clustering of clinical variables across cohorts (Seymour et al., 2019)	Alpha: fewer abnormalities; beta: older/renal; gamma: inflammation/pulmonary; delta: liver dysfunction/shock	Vital signs, laboratories, organ dysfunction and comorbidities	Can balance or stratify trials	Externally evaluated clinical phenotypes	No prospective differential heparin effect
SRS/MARS transcriptomic endotypes	Whole-blood gene-expression classes ( Davenport et al., 2016; Scicluna et al., 2017 )	Variable innate activation, adaptive suppression, endotoxin tolerance and outcome risk	Gene-expression classifier; immune dysfunction scores	May separate immune-active from immunosuppressed states	Prospective observational cohorts	Platform and timing dependent; no validated heparin-response association
NET/histone-high state	Research biomarker enrichment ( Fuchs et al., 2010; Iba et al., 2015 )	Excess extracellular chromatin and cationic histone burden	cfDNA, MPO-DNA, citrullinated histone H3, circulating histones	Mechanistically aligned with polyanion-mediated neutralization	Preclinical and prognostic evidence	Assay standardization and treatment thresholds absent
Endotheliopathic/glycocalyx-shedding state	Research biomarker enrichment ( Uchimido et al., 2019; Eustes et al., 2021 )	Barrier failure, vascular leak, heparan sulfate loss	Syndecan-1, heparan sulfate, heparanase activity, angiopoietin-2	Mechanistically aligned with heparanase inhibition and surface protection	Biomarker and preclinical evidence	Markers are not heparin-specific and may reflect severity rather than treatable mechanism

cfDNA, cell-free DNA; DIC, disseminated intravascular coagulation; FDP, fibrin degradation products; ISTH, International Society on Thrombosis and Haemostasis; MARS, molecular diagnosis and risk stratification of sepsis; MIMIC, Medical Information Mart for Intensive Care; MPO, myeloperoxidase; NET, neutrophil extracellular trap; PT-INR, prothrombin time international normalized ratio; SIC, sepsis-induced coagulopathy; SOFA, Sequential Organ Failure Assessment; SRS, sepsis response signature.

**FIGURE 1:**
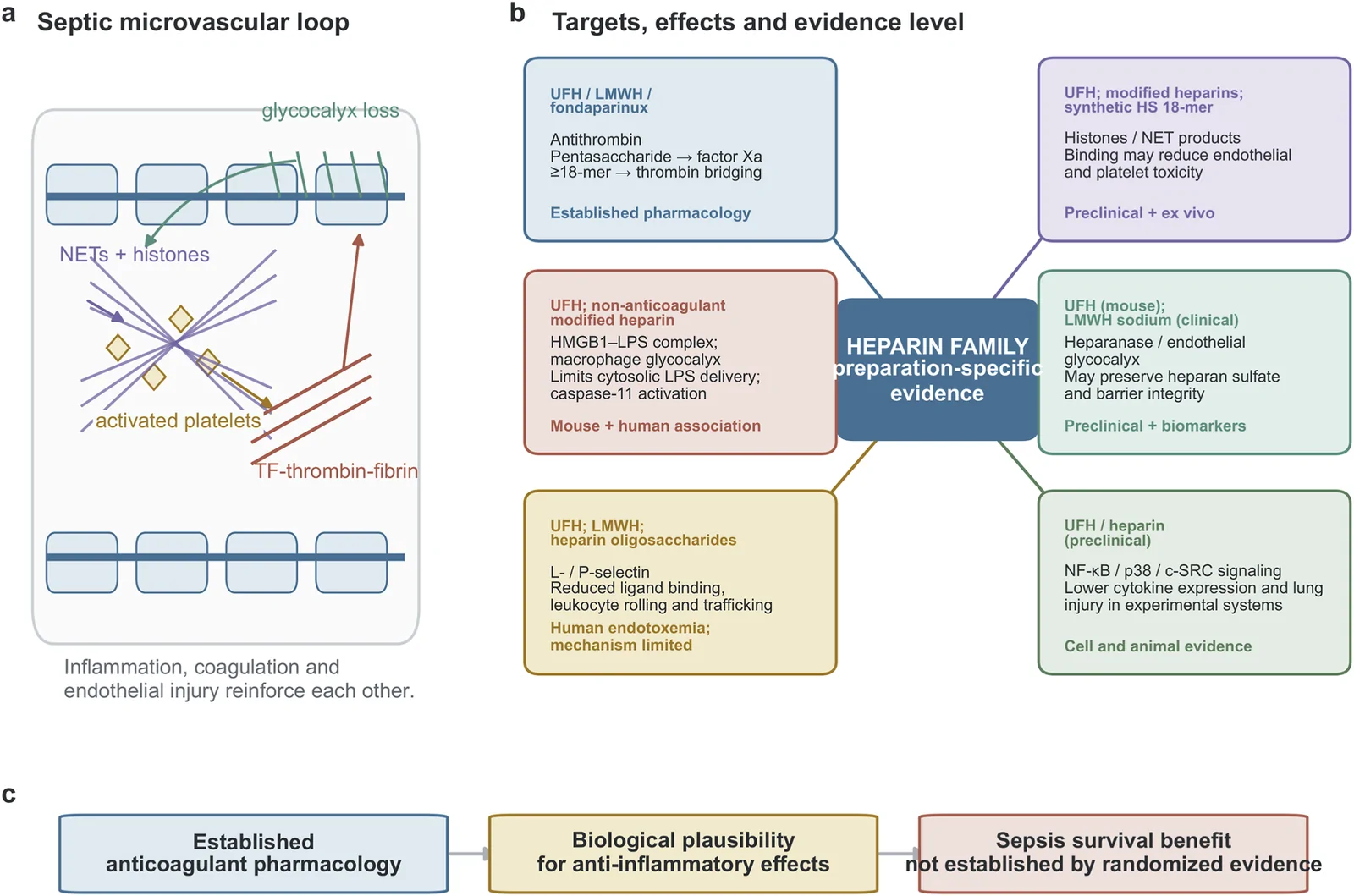
Heparin-family targets in septic immunothrombosis and the corresponding evidence hierarchy. **(a)** Excessive NET formation, platelet activation, tissue factor-thrombin-fibrin signaling, and glycocalyx loss reinforce microvascular injury. **(b)** Each target box specifies the preparation studied, including UFH, LMWH, modified non-anticoagulant heparin, or a synthetic heparan sulfate oligosaccharide; the preparation and strength of evidence vary by target. **(c)** Biological plausibility for non-anticoagulant effects does not establish a sepsis survival benefit. AT, antithrombin; HMGB1, high-mobility group box 1; LMWH, low-molecular-weight heparin; LPS, lipopolysaccharide; NET, neutrophil extracellular trap; UFH, unfractionated heparin.

**FIGURE 2:**
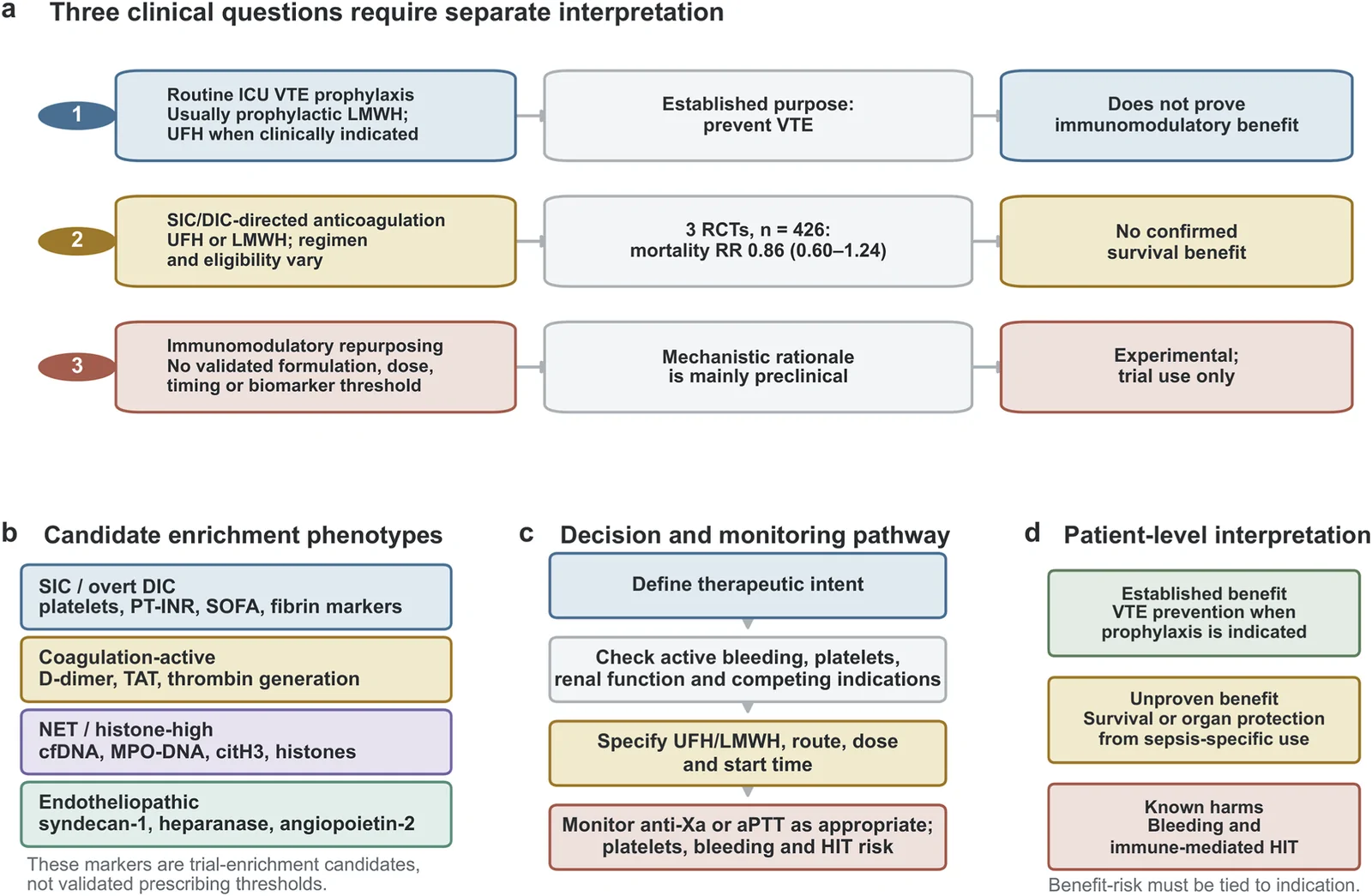
Separating the three clinical questions and the corresponding interpretation pathway. **(a)** Routine VTE prophylaxis, coagulopathy-directed anticoagulation, and experimental immunomodulatory repurposing are distinct questions and the evidence for one does not transfer to another. **(b)** Candidate enrichment phenotypes for trial design. **(c)** Decision and monitoring pathway anchored on a prespecified therapeutic intent. **(d)** Patient-level interpretation: benefit and risk must be tied to the indication. DIC, disseminated intravascular coagulation; SIC, sepsis-induced coagulopathy; VTE, venous thromboembolism.

## 2 The pathophysiological foundation

### 2.1 Cellular drivers of immunothrombosis

In sepsis, immunothrombosis is driven by a complex interplay between innate immune cells and the coagulation system, with neutrophil extracellular traps (NETs) and platelet-leukocyte aggregates (PLAs) serving as central pathological hubs (Retter et al., 2025; Wienkamp et al., 2022; Iba et al., 2023b; Liao et al., 2025). As primary cellular responders of innate immunity, neutrophils can undergo NETosis, a specialized form of cell death that releases web-like structures composed of decondensed DNA, histones, and antimicrobial proteins such as myeloperoxidase (MPO) and neutrophil elastase (NE) (Zhang H et al., 2023; Islam and Takeyama, 2023; Denning et al., 2019). Although initially protective, excessive or dysregulated NET formation creates a potent procoagulant scaffold that promotes fibrin deposition and traps both pathogens and host cells (Retter et al., 2025; Wienkamp et al., 2022; Zhang H et al., 2023). These structures activate the contact pathway of coagulation and induce endothelial damage, leading to tissue factor (TF) production and degradation of the protective glycocalyx (Retter et al., 2025; Zhang H et al., 2023; Islam and Takeyama, 2023). The relationship between NETs and STING signaling requires a more specific statement than is often made. In a murine model of sepsis-associated lung injury, NET exposure activated STING signaling in endothelial cells, increased tissue factor expression, and involved endothelial Toll-like receptor 2; disrupting either NETs or STING reduced inflammation and coagulation (Zhu et al., 2023). A separate study showed that platelet-intrinsic STING enhanced platelet activation and granule secretion during septic thrombosis (Yang M. et al., 2023). Taken together these findings do not demonstrate that NETs directly activate platelet STING, and neither pathway has been validated as heparin-responsive in patients. This interplay is reinforced by PLAs, particularly platelet-neutrophil and platelet-monocyte conjugates, which are hallmarks of the dysregulated host response and facilitate leukocyte recruitment to sites of vascular injury (Wienkamp et al., 2022; Schrottmaier et al., 2020). The clinical associations vary by setting: a small severe-sepsis cohort found higher early platelet-leukocyte conjugate levels among survivors, whereas platelet hyperreactivity and innate immune activation were associated with greater severity in a separate COVID-19 cohort (Soriano et al., 2005; Jakobs et al., 2022). Neither observation establishes a causal treatment target or a response to heparin. The resulting NET-driven response creates a self-amplifying loop of thromboinflammation in which cell-free DNA and NET-associated histones mediate direct cytotoxicity, ultimately culminating in microcirculatory collapse and life-threatening multiorgan failure (Retter et al., 2025).

### 2.2 Molecular triggers and inflammatory cascades

The transition from protective immunity to pathological immunothrombosis in sepsis is orchestrated by a molecular network in which the monocyte-endothelial tissue factor (TF) axis links inflammation to coagulation initiation. Within the septic microenvironment, inflammatory stimuli such as lipopolysaccharide (LPS) and paracrine signals from activated endothelial cells drive monocytes to express TF, the key initiator of the extrinsic coagulation pathway. TF expression is significantly higher in patients with established sepsis than in non-septic critically ill patients, particularly within intermediate and non-classical monocyte subsets (Musgrave et al., 2023). This crosstalk is further amplified by pathogen-associated molecular patterns (PAMPs) and host-derived DAMPs, which signal through pattern-recognition receptors such as TLR4 to trigger a systemic cytokine storm (Denning et al., 2019). Central to this cascade is high-mobility group box 1 (HMGB1), a non-histone chromosomal protein that functions as a potent pro-inflammatory cytokine after extracellular release (Ghosh and Ghosh, 2020; Yuan et al., 2024). Extracellular HMGB1 engages receptors such as RAGE and TLR2/4 to stimulate immune cells, establishing a positive-feedback loop that exacerbates cellular injury and organ damage (Yuan et al., 2024; Denning et al., 2019).

Concurrently, NETs release decondensed chromatin fibers containing extracellular histones and cell-free DNA (cfDNA). In experimental systems, NETs provide a scaffold for platelet adhesion and fibrin deposition, while histones can directly activate platelets and damage endothelial cells (Fuchs et al., 2010; Gould et al., 2015; Iba et al., 2015). Clinical studies summarized by Li et al. (2021) associate circulating histone concentrations with sepsis severity and adverse outcomes, but do not establish that histone neutralization improves patient outcomes. Ligi et al. (2022) compared monocyte distribution width in sepsis and COVID-19 and showed that histone exposure altered monocyte morphology in healthy whole blood *in vitro*. This supports a potential contribution of histones to cellular inflammatory changes, rather than a demonstrated therapeutic effect of heparin. These molecular interactions provide a biological basis for a reinforcing relationship between inflammation and coagulation (Denning et al., 2019).

### 2.3 Endothelial failure and the glycocalyx

Sepsis involves interacting inflammatory, coagulation, and endothelial pathways (Iba et al., 2023b). A central component of endothelial injury is degradation of the glycocalyx, a surface layer containing proteoglycans such as syndecan-1 and glycosaminoglycan chains such as heparan sulfate (Uchimido et al., 2019; Oshima et al., 2021). Experimental and clinical literature implicates inflammatory mediators, oxidative stress, heparanase, and other shedding enzymes in glycocalyx disruption (Iba and Levy, 2019; Uchimido et al., 2019). Loss of heparan sulfate impairs a surface that supports antithrombin activity, while endothelial injury also disturbs protective thrombomodulin and protein C pathways. These interacting changes favor a procoagulant endothelial state (Iba and Levy, 2019; Mussbacher et al., 2019).

The consequences of this endotheliopathy include altered vascular tone, microvascular thrombosis, and increased permeability, which contribute to capillary leakage and tissue edema (Oshima et al., 2021; Iba et al., 2025; Kravitz et al., 2024). Circulating glycocalyx components such as syndecan-1 and heparan sulfate have been investigated as markers of endothelial injury and disease severity (Uchimido et al., 2019). In patients with severe sepsis and respiratory failure, angiopoietin-2 was associated with severe acute kidney injury (Yu et al., 2021). Such prognostic associations do not validate these biomarkers as measures of response to heparin. Glycocalyx preservation is therefore a mechanistically motivated research strategy whose clinical value in sepsis requires prospective evaluation (Iba and Levy, 2019; Kravitz et al., 2024).

## 3 The pleiotropic mechanisms of heparin

### 3.1 Canonical mechanism

The anticoagulant activity of heparin is primarily mediated through its interaction with antithrombin, a key plasma inhibitor of coagulation factors. This canonical mechanism depends on a specific pentasaccharide sequence within the heparin polymer that binds antithrombin with high affinity. The high-affinity motif can be written as GlcNAc6S-GlcA-GlcNS3S6S-IdoA2S-GlcNS6S, in which the 3-O-sulfate group on the central glucosamine residue is the critical determinant of binding (Mulloy et al., 2016; Hogwood et al., 2023b). Binding induces a conformational change in antithrombin that is sufficient on its own to accelerate the inhibition of factor Xa. Inhibition of thrombin, however, requires more than the conformational change: the heparin chain must also bridge antithrombin and thrombin simultaneously, which demands a chain of at least approximately 18 saccharide units. The pentasaccharide alone is therefore not sufficient for a full anti-factor IIa effect, and this length dependence explains why fondaparinux, which acts entirely through antithrombin, inhibits factor Xa without meaningful anti-factor IIa activity, why LMWHs have higher anti-Xa to anti-IIa ratios than UFH, and why only UFH reliably prolongs the activated partial thromboplastin time (Mulloy et al., 2016; Hogwood et al., 2023b). The same length dependence governs reversal: protamine sulfate neutralizes UFH almost completely, neutralizes LMWH only partially, and does not neutralize fondaparinux, which is one practical reason UFH is sometimes preferred when rapid discontinuation or pharmacological reversal may be required (Mulloy et al., 2016; Hogwood et al., 2023b). Although this antithrombin-dependent pathway has historically defined the clinical utility of heparin, the structural complexity of the molecule enables a broader spectrum of biochemical interactions. This insight has driven the development of synthetic heparin mimetics and low-molecular-weight derivatives designed to refine these inhibitory pathways for acute clinical use (Mulloy et al., 2016).

### 3.2 Anti-inflammatory properties

Beyond its established anticoagulant action, heparin has shown anti-inflammatory effects in experimental systems; these findings do not establish clinical efficacy in patients with sepsis (Yu et al., 2024; Li and Ma, 2017). One frequently reported mechanism is reduced NF-kappa-B pathway activation, with lower release of pro-inflammatory cytokines including IL-1-beta, IL-6, and TNF-alpha (Li et al., 2013; Zhao et al., 2012). In an LPS-induced rat lung-injury model, UFH lowered IL-1-beta and IL-6 expression and reduced NF-kappa-B activation, and in a separate murine model heparin reduced pulmonary p38 phosphorylation, NF-kappa-B expression, c-SRC phosphorylation, cytokine levels, edema, and lethality (Li et al., 2013; Zhao et al., 2012). The mechanism by which a large, highly charged polysaccharide that does not readily cross the plasma membrane produces these intracellular changes is not established. Because heparin binds LPS, HMGB1, histones, and selectins in the extracellular space, the most parsimonious explanation is upstream sequestration of these ligands and consequent reduction of TLR4-and RAGE-dependent signaling, rather than direct engagement of an intracellular target in the NF-kappa-B cascade (Tang et al., 2021; Wang et al., 2002). NF-kappa-B inhibition is therefore best regarded as a downstream preclinical signature of reduced receptor-level stimulation, not as a demonstrated intracellular drug action, and it has not been shown in septic patients.

In biochemical and cell-based systems, heparin binds cytotoxic cationic proteins, particularly extracellular histones released during NET formation and cell death (Li et al., 2021; Longstaff et al., 2016). Heparins reduced histone-mediated cytotoxicity *in vitro* and improved survival in a rat model of histone-induced organ dysfunction (Iba et al., 2015). Huckriede et al. (2025) further showed that heparin enhanced neutrophil-elastase-mediated histone proteolysis and reduced histone-mediated cytotoxicity *in vitro*. Proteolysis of histones H3 and H4 did not, however, reduce TLR4 activation in the assay used. These findings identify experimentally demonstrated interactions; they do not establish a reduction in thromboembolic events or mortality in patients.

Tang et al. (2021) demonstrated in murine experimental systems that heparin and a chemically modified non-anticoagulant heparin inhibited HMGB1-LPS binding and prevented heparanase-dependent degradation of the macrophage glycocalyx. These actions limited cytosolic LPS delivery in macrophages and reduced caspase-11 activation. The study did not test the same mechanism in vascular endothelial cells or in patients with sepsis. Together with the histone and cytokine findings, these results provide a biological rationale for further investigation of heparin as a potential multitarget immunomodulatory agent. They do not establish clinical efficacy or support sepsis-specific immunomodulatory use outside a clinical trial.

### 3.3 Endothelial and glycocalyx protection

The endothelial glycocalyx (eGC), a complex gel-like layer composed of proteoglycans, glycoproteins, and heparan sulfate (HS), is a critical regulator of vascular homeostasis, microvascular tone, and barrier integrity (Oshima et al., 2021; Uchimido et al., 2019). During sepsis progression, systemic induction of sheddases, most notably heparanase (HPSE), results in enzymatic cleavage of HS side chains and shedding of structural components such as syndecan-1 (SDC-1) (Uchimido et al., 2019; Iba and Levy, 2019). Heparanase is the only known mammalian endoglycosidase that cleaves heparan sulfate. In humans it is the product of a single gene, HPSE, and is synthesized as a latent proenzyme that requires cathepsin L-mediated processing in the lysosome before it becomes active. The principal cellular sources relevant to sepsis are activated platelets, neutrophils, monocytes and macrophages, mast cells, and the endothelium itself; expression is further induced in endothelial cells by pro-inflammatory cytokines and by reactive oxygen species (Uchimido et al., 2019; Eustes et al., 2021; Drost et al., 2022). This degradation leads to vascular hyperpermeability, dysregulated vasodilation, and the capillary leak syndrome characteristic of critical illness (Oshima et al., 2021; Uchimido et al., 2019).

Heparin and selected derivatives can inhibit HPSE in experimental systems, providing a rationale for investigating glycocalyx preservation (Hogwood et al., 2023a; Liao et al., 2023). Tang et al. (2021) reported two related actions in murine systems: heparin disrupted HMGB1-LPS binding, and heparin prevented heparanase-dependent degradation of the glycocalyx on the surface of macrophages. Both actions limited delivery of LPS to the cytosol and reduced caspase-11 activation. The glycocalyx in those experiments belonged to macrophages rather than to vascular endothelial cells, so the finding supports a mechanism of innate immune-cell LPS uptake and is distinct from preservation of the endothelial glycocalyx, which concerns vascular barrier biology (Tang et al., 2021). In patients with COVID-19, plasma HPSE activity was associated with markers of endothelial glycocalyx damage (Drost et al., 2022). In a separate observational cohort, prophylactic LMWH use in non-ICU patients with COVID-19 was associated with lower plasma HPSE activity (Buijsers et al., 2020). These findings do not establish a causal treatment effect or clinical benefit in sepsis, and the Buijsers cohort did not evaluate LMWH as a sepsis-specific immunomodulatory treatment. In septic patients with acute gastrointestinal injury, LMWH treatment lowered serum SDC-1 and HPSE levels and reduced acute gastrointestinal injury grade, although 28-day survival did not differ (Chen et al., 2023). These are biomarker and organ-function endpoints in a single-center trial and do not establish a survival benefit.

Evidence from non-sepsis experimental models provides additional mechanistic context. In rat lung-transplantation and *ex vivo* lung-perfusion models, heparin or N-acetyl heparin inhibited heparanase, preserved the endothelial glycocalyx, and reduced edema, inflammatory-cell infiltration, and vascular leakage (Noda et al., 2021). Mitra et al. (2025) studied heparin combined with sphingosine-1-phosphate (S1P) in cultured endothelial cells and a mouse disturbed-flow model, reporting glycocalyx restoration and improved endothelial function. In diabetic mice, the heparanase inhibitor OVZ/HS-1638 preserved glycocalyx structure and reduced retinal and glomerular permeability (Gamez et al., 2024). These studies involve distinct preparations and disease models; they do not establish efficacy, dosing, or safety for glycocalyx-directed treatment of patients with sepsis.

### 3.4 Modulation of leukocyte trafficking

In biochemical and preclinical studies, heparin and its derivatives can disrupt the selectin-mediated adhesion cascade, a critical step in leukocyte recruitment to inflamed tissues. Heparin oligosaccharides competitively bind both L- and P-selectin and inhibit leukocyte rolling and adhesion to the vascular endothelium, an effect that requires 6-O-sulfation of glucosamine residues (Wang et al., 2002). This inhibitory capacity is strongly influenced by chain length and sulfation pattern: whereas UFH and specific LMWHs such as tinzaparin attenuate selectin-mediated interactions, smaller synthetic saccharides such as fondaparinux showed little selectin inhibition in the non-sepsis metastasis experiments cited here (Stevenson et al., 2005). The human evidence cited here comes from an endotoxemia challenge in healthy volunteers, in which UFH attenuated the lipopolysaccharide-induced fall in neutrophil surface L-selectin, with a less prominent effect for LMWH; that study did not establish whether the finding reflected reduced shedding, altered internalization, or changed cell trafficking (Derhaschnig et al., 2003). Combined anticoagulant and antiselectin strategies prevented lethal intravascular coagulation in a murine model (Norman et al., 2003). No clinical study has tested whether selectin blockade by any heparin preparation changes outcomes in sepsis.

The HETRASE trial is often cited in this context, but it tested UFH as a sepsis treatment and was null; it does not provide evidence that selectin-directed effects translate into clinical benefit (Jaimes et al., 2009). The selectin data in humans come from an endotoxemia challenge study in healthy volunteers rather than from patients with sepsis (Derhaschnig et al., 2003). Effects on leukocyte trafficking therefore remain a mechanistic rationale for further study rather than a demonstrated therapeutic action.

## 4 Repurposing heparin in the clinical context

### 4.1 Historical evidence and challenges

Clinical investigations of heparin in sepsis have produced heterogeneous results. Before those results are compared, three distinct clinical questions must be separated, because evidence about one does not transfer to another: routine VTE prophylaxis in critically ill patients, anticoagulation directed at SIC or overt DIC, and experimental immunomodulatory repurposing. Much of the apparent conflict in the literature arises from treating these as a single question. The HETRASE trial randomized 319 emergency-department patients with sepsis to placebo or UFH 500 U/hour for 7 days. UFH did not improve the prespecified length-of-stay or multiple-organ-dysfunction outcomes, and 28-day mortality was 14% with UFH versus 16% with placebo (p = 0.652); subgroup analyses were also negative (Jaimes et al., 2009). HETRASE used a fixed infusion without titration to a prespecified aPTT or anti-Xa target, and the control-group mortality was lower than in many sepsis trials. These are features of the trial rather than demonstrated causes of the null result, and it has not been shown that a higher or titrated dose would have produced benefit; the earlier suggestion that the regimen was simply ineffective is not supported by data (Barochia et al., 2009). A small prospective study randomized 37 patients with early DIC to saline plus sepsis care or UFH 70 U/kg/day for 5–7 days, and found improvement in selected coagulation measures but no significant difference in 28-day mortality (31.8% versus 40.0%; p = 0.434) (Liu et al., 2014). Importantly, the most recent systematic review restricted to randomized trials pooled three trials and 426 patients and found no significant mortality reduction with heparin (risk ratio [RR] 0.86, 95% confidence interval [CI] 0.60–1.24); the sepsis-associated DIC subgroup was likewise inconclusive (n = 109; RR 0.84, 95% CI 0.51–1.38), and although pooled bleeding was lower (RR 0.49, 95% CI 0.24–0.99), the small and heterogeneous evidence base precludes a definitive safety advantage (Totoki et al., 2024). Earlier meta-analyses reached more favorable but less secure conclusions: Wang et al. (2014) reported lower 28-day mortality (odds ratio [OR] 0.656, 95% CI 0.562–0.765; n = 3,482), but several included datasets represented concomitant prophylactic or catheter-flush heparin exposure within trials of other anticoagulants rather than randomized heparin treatment, and Zarychanski et al. (2015) found an RR for death of 0.88 (95% CI 0.77–1.00) with imprecise major-bleeding estimates (RR 0.79, 95% CI 0.53–1.17). Taken together, the randomized evidence does not establish a survival benefit, and the balance of randomized and observational findings supports uncertainty rather than efficacy. Table 1 places these studies on common fields so that therapeutic purpose, patient definition, preparation, dose, timing, comparator, effect estimate, and bleeding can be compared directly.

### 4.2 Identifying responders

Any discussion of responders depends on how the candidate population is defined, so the two operative definitions are stated explicitly here. SIC is scored by the ISTH criteria using platelet count, prothrombin time-INR, and the SOFA score, with a total score of at least four and a combined platelet-count and prothrombin-time score greater than two; overt DIC is scored by the ISTH algorithm using platelet count, a fibrin-related marker, prothrombin time, and fibrinogen, with a total of at least 5 (Iba et al., 2017; Iba et al., 2023a; Iba et al., 2023b). SIC identifies an earlier, compensated phase, whereas overt DIC identifies a late consumptive phase, which is why the two should not be pooled. Current evidence indicates that patients meeting SIC or DIC criteria are the subgroups in which anticoagulant intervention has most often been studied (Iba et al., 2023b). Retrospective cohorts report associations between UFH exposure and survival in patients with SIC, but these findings do not establish a reproducible response threshold or a causal benefit at a particular SIC score (Peng et al., 2022; Huang et al., 2023). Latent class analysis in MIMIC-IV identified data-derived subphenotypes in which lower 28-day mortality during heparin exposure was confined to Class 2, a group characterized by severe coagulopathy, high lactate, and profound organ dysfunction, with no comparable signal in other classes (Cai et al., 2023). Because that class was both derived and evaluated in the same database, it is a hypothesis for prospective testing rather than a validated responder definition. The control treatment differed across these studies and this affects interpretation: HETRASE used placebo, Liu et al. used saline plus standard sepsis care, Chen et al. used subcutaneous saline plus conventional treatment, Weng et al. used conventional treatment alone, and the observational cohorts compared patients who received no early heparin, which is not a randomized comparator (Jaimes et al., 2009; Liu et al., 2014; Chen et al., 2023; Weng et al., 2021; Zou et al., 2022). Beyond conventional scoring, candidate enrichment biomarkers include platelet count and prothrombin time-INR for coagulopathy; D-dimer, fibrin degradation products, thrombin-antithrombin complexes, and prothrombin fragment 1 + 2 for thrombin generation; cell-free DNA, MPO-DNA complexes, citrullinated histone H3, and circulating histones for NET burden; and syndecan-1, heparan sulfate, heparanase activity, and angiopoietin-2 for endothelial and glycocalyx injury (Uchimido et al., 2019; Eustes et al., 2021; Li et al., 2021; Yu et al., 2021). None of these has a validated threshold for selecting or dosing heparin, and Table 2 sets out their current evidence status.

Reports that heparin corrects sepsis-induced platelet depletion and improves DIC resolution require the same care. A broader synthesis of anticoagulant rather than heparin-specific therapy found that in sepsis-associated DIC, across nine studies and 17,968 patients, anticoagulation was not associated with a statistically significant reduction in all-cause mortality (RR 0.89, 95% CI 0.72–1.10) or a statistically significant increase in serious bleeding (RR 1.27, 95% CI 0.77–2.09), although the DIC resolution rate was higher (OR 2.62, 95% CI 1.54–4.45); because that analysis pooled several anticoagulant classes and included non-randomized studies, it cannot isolate a heparin-specific effect (Qi et al., 2023). Improved DIC resolution is therefore not interchangeable with improved survival. Three features of the observational literature deserve explicit statement because they can generate apparent benefit in the absence of any true effect. First, confounding by contraindication: patients who are bleeding, thrombocytopenic, or expected to die imminently are less likely to receive heparin, so the treated group is systematically less sick in ways that measured covariates capture incompletely. Second, immortal time bias: when exposure is defined by treatment received within a window such as the first 24 or 48 h, a patient must survive until treatment begins; assigning that pre-treatment survival time to the exposed group can favor treatment unless exposure timing is handled appropriately, for example, using a time-varying or suitable landmark analysis. Third, time-varying confounding: dose, indication, and continuation all change in response to the evolving clinical course, so a single baseline exposure label misrepresents the actual treatment. Matched analyses reduce but do not remove these problems, and several of the cited cohorts report longer ICU and hospital stay in treated patients, which is consistent with residual imbalance in survival opportunity (Zou et al., 2022; Peng et al., 2022; Huang et al., 2023). Identifying responders will therefore require prospective testing in patients with severe coagulation activation rather than further retrospective association studies (Yu et al., 2024).

### 4.3 Heparin formulations

Discussion of formulation must be tied to therapeutic purpose, because the evidence base differs across these clinical purposes. For routine VTE prophylaxis in critically ill patients, typical regimens are UFH 5,000 U subcutaneously every 8–12 h or enoxaparin 40 mg subcutaneously once daily, with dose adjustment for renal impairment and body weight; LMWH is generally preferred in this setting because of more predictable pharmacokinetics, once-daily administration, and a lower risk of heparin-induced thrombocytopenia (Beitland et al., 2015). A systematic review of randomized ICU thromboprophylaxis trials found fewer deep vein thromboses and a favorable composite net clinical outcome with LMWH compared with UFH, without a statistically significant mortality difference (Beitland et al., 2015). This is a thromboprophylaxis recommendation and it cannot be extrapolated to sepsis-directed anticoagulation or to immunomodulatory repurposing. For coagulopathy-directed treatment the regimens studied varied in preparation, route, and intensity: UFH 500 U/hour by continuous infusion in HETRASE, UFH 70 U/kg/day in early DIC, heparin sodium 50 mg/day by continuous pump as a comparator arm, and LMWH sodium 4,000 U subcutaneously once daily in sepsis-associated acute gastrointestinal injury (Jaimes et al., 2009; Liu et al., 2014; Weng et al., 2021; Chen et al., 2023). Earlier favorable mortality estimates pooled heterogeneous treatment purposes and exposures and should be interpreted with the design limitations described in Section 4.1 (Wang et al., 2014; Zarychanski et al., 2015). Recent experimental work suggests context-specific differences between preparations; UFH inhibited Drp1-mediated mitochondrial quality imbalance and reduced vascular leakage in a preclinical model, an effect not established for LMWH (Liu R. et al., 2024). No randomized comparison has tested UFH against LMWH for a sepsis-specific immunomodulatory indication, so no preference can be stated for that purpose. A practical consequence for trial design follows from this separation: because guideline-recommended VTE prophylaxis will be given to most enrolled patients, a sepsis-specific treatment trial must standardize that background prophylaxis in both arms, so that the randomized comparison isolates the sepsis-directed intervention rather than the presence or absence of prophylaxis (Evans et al., 2021).

Non-anticoagulant heparinoids represent a therapeutic frontier aimed at decoupling anti-inflammatory and histone-neutralizing properties from systemic anticoagulation in order to minimize hemorrhagic risk. Structurally they retain the sulfated, polyanionic glycosaminoglycan backbone of heparin but are altered so that the antithrombin-binding pentasaccharide is disrupted or removed. The principal strategies are N-desulfation with N-acetylation, selective O-desulfation such as 2-O- and 3-O-desulfation, glycol splitting of non-sulfated uronic acid residues, and defined chemoenzymatic synthesis of homogeneous oligosaccharides. The resulting molecules have greatly reduced affinity for antithrombin and therefore little anti-Xa or anti-IIa activity, while retaining sufficient charge and chain length to bind histones, HMGB1-LPS complexes, heparanase, or selectins (Hogwood et al., 2023a; Hogwood et al., 2023b; Shute, 2023). Because each modification alters several binding properties at once, retained anti-inflammatory activity has to be demonstrated rather than assumed. The modified non-anticoagulant heparin used by Tang et al. (2021) and the defined heparan sulfate 18-mer studied by Liao et al. (2023) are proof-of-concept tools rather than clinically validated sepsis therapies (Tang et al., 2021; Liao et al., 2023).

## 5 Safety, monitoring, and implementation barriers

### 5.1 Hemorrhagic risks

The bleeding risk of heparin must be evaluated in relation to its clinical indication, preparation, dose, and patient population. Early meta-analyses reported favorable mortality estimates, but their heterogeneous treatment purposes, study designs, and imprecise safety estimates limit causal interpretation (Wang et al., 2014; Zarychanski et al., 2015). The randomized-trial meta-analysis discussed in Section 4.1 did not establish a survival benefit; its lower pooled bleeding estimate was based on a small evidence base and does not establish a general safety advantage (Totoki et al., 2024). Histone neutralization and modulation of inflammatory signaling are supported mainly by biochemical and preclinical studies (Iba et al., 2015; Tang et al., 2021). These findings do not establish a clinically safe balance in which immunomodulatory heparin treatment reduces MODS or ICU stay without major hemorrhage. A lack of statistically significant bleeding excess in an individual study is not proof that clinically important bleeding risk has been excluded.

Additional randomized safety information comes from the historical XPRESS prophylaxis setting. XPRESS randomized 1,994 patients with severe sepsis who were receiving drotrecogin alfa (activated) to UFH 5,000 U every 12 h, enoxaparin 40 mg once daily, or placebo during the 96-h infusion. No statistically significant mortality difference was established. Any bleeding during days 0–six was more frequent with heparin than with placebo (10.8% versus 8.1%; p = 0.049), whereas serious bleeding was similar (2.3% versus 2.5%; p = 0.72) (Levy et al., 2009). Because the comparison was made in a historical drotrecogin alfa context and tested prophylactic dosing, it informs prophylaxis safety rather than sepsis-specific immunomodulation. These results show that safety findings depend on the bleeding endpoint and treatment context, and do not establish safety for immunomodulatory dosing (Zarychanski et al., 2015). Whether efficacy depends on patient selection and dosing strategy remains an open question rather than a demonstrated finding: fixed-rate infusions have not shown benefit in populations with low baseline mortality, but no randomized comparison has established that titrated therapeutic dosing performs better in more severely ill patients (Barochia et al., 2009). Targeted evaluation in phenotypes characterized by SIC or overt DIC is therefore a reasonable trial strategy rather than a validated approach, and biomarkers of severe coagulation activation will be needed to test it prospectively (Guo et al., 2022).

Further development of heparin for sepsis-specific immunomodulation requires prospective trials that prespecify the formulation, dose, treatment timing, background VTE prophylaxis, and eligible phenotype. Such trials should evaluate patient-centered outcomes alongside target engagement, major bleeding, and HIT. Whether a clinically useful balance between immunomodulatory activity and hemostatic safety can be achieved remains an open question (Totoki et al., 2024; Hogwood et al., 2023a).

### 5.2 Heparin-induced thrombocytopenia (HIT)

Heparin-induced thrombocytopenia (HIT) is a severe immune-mediated prothrombotic complication triggered by antibodies directed against the platelet factor 4 (PF4)-heparin complex. Although HIT occurs in 0.2%–5.0% of heparin-exposed patients, its incidence in the intensive care unit (ICU) is relatively low, at approximately 0.39%–0.5% (Gupta et al., 2015; Selleng and Selleng, 2016). In patients with sepsis, however, HIT presents a major diagnostic dilemma because thrombocytopenia is often driven by competing etiologies such as SIC, DIC, or drug-induced marrow suppression (Selleng and Selleng, 2016; Gupta et al., 2015). Clinical pretest probability should be assessed because a positive PF4-heparin immunoassay does not by itself establish platelet-activating HIT antibodies. When clinical probability is intermediate or high, heparin should be discontinued and non-heparin anticoagulation, such as argatroban or bivalirudin, considered while confirmatory testing and bleeding risk are assessed (Gupta et al., 2015; Selleng and Selleng, 2016). In patients with sepsis-associated MODS, argatroban dosing must be carefully titrated and may need to be reduced to as little as one-eighth of the standard dose because of impaired clearance and high overdose risk (Saugel et al., 2010).

Monitoring evidence for argatroban should be distinguished from monitoring of heparin. In a prospective study of 22 critically ill patients receiving argatroban, diluted thrombin time correlated more closely with measured drug concentrations than aPTT; viscoelastic ecarin clotting time also provided information about drug exposure (Heubner et al., 2024). These assay comparisons support further evaluation of monitoring strategies but do not themselves demonstrate improved clinical outcomes. A case report also illustrates that adrenal insufficiency can complicate HIT-related shock (Mongardon et al., 2007).

### 5.3 Monitoring beyond aPTT

UFH anticoagulation is commonly monitored with aPTT or anti-Xa activity. In critical illness, acute-phase changes and variations in coagulation factors, including factor VIII, can contribute to disagreement between these measurements (Hogwood et al., 2023b; Lardinois et al., 2022). An aPTT that suggests insufficient anticoagulation may therefore not always indicate low heparin activity. These limitations require interpretation in the clinical and assay context; neither aPTT nor anti-Xa is a validated measure of heparin’s proposed immunomodulatory effects.

Point-of-care (POC) viscoelastic testing, including rotational thromboelastometry (ROTEM) and thromboelastography (TEG), provides complementary information about clot formation and fibrinolysis (Levi, 2017). An observational ICU study examined the association between ROTEM clotting time and aPTT during heparin treatment (Najafi et al., 2015). Correlation between assays does not establish interchangeability, superior dose titration, or improved patient outcomes. These tests should therefore be discussed as potential adjuncts requiring indication-specific validation, rather than as validated replacements for standard UFH monitoring.

Experimental findings also motivate investigation of biomarkers beyond conventional anticoagulation assays. For example, UFH attenuated histone-induced pulmonary syndecan-1 loss in a mouse model (Fu et al., 2022), while histone neutralization has been demonstrated in cell and animal experiments (Iba et al., 2015). Syndecan-1, circulating histones, and HPSE activity could be evaluated as exploratory pharmacodynamic measures in future trials. They currently lack validated response thresholds, specificity for heparin effects, and evidence that biomarker-guided dosing improves clinical outcomes (Uchimido et al., 2019; Yu et al., 2024).

In the 35-patient ICU study by Lardinois et al. (2022), POC-aPTT results were available in less than 5 minutes, but agreement with laboratory aPTT and anti-Xa measurements was poor. The investigators cautioned against applying laboratory-aPTT nomograms to the POC assay. Integrating rapid coagulation testing with research biomarkers is a future validation task; a resulting improvement in the therapeutic index of heparin in septic shock has not been demonstrated.

## 6 Future directions and emerging strategies

### 6.1 Synthetic heparin mimetics

Synthetic and chemically modified heparin mimetics are being investigated to retain selected biological interactions while reducing antithrombin-dependent anticoagulation (Hogwood et al., 2023a; Hogwood et al., 2023b). Biochemical and animal studies identified glucosamine 6-O-sulfation as important for L- and P-selectin inhibition (Wang et al., 2002). Modifications such as selective desulfation or N-desulfation with N-acetylation alter several binding properties, so retained selectin inhibition, histone binding, and HPSE inhibition must be established separately for each preparation (Hogwood et al., 2023b; Shute, 2023). Reduced anticoagulant activity in an assay does not establish freedom from hemorrhagic or other adverse effects in patients with sepsis.

Chemoenzymatic synthesis and experimental delivery platforms offer ways to investigate structurally defined heparin-related compounds (Hogwood et al., 2023a; Sultana and Kamihira, 2024). A defined heparan sulfate 18-mer was evaluated as a multitarget agent in experimental sepsis, providing proof of concept for a specific oligosaccharide rather than a clinically validated therapy (Liao et al., 2023). Anticoagulant activity and interactions with inflammatory targets vary across these compounds and should not be assumed to reproduce the effects of UFH or LMWH.

Clinical translation will require reproducible manufacturing, pharmacokinetic and target-engagement studies, and prospective evaluation of efficacy and safety. The available biochemical and preclinical evidence supports this research program but does not yet define a sepsis treatment strategy (Hogwood et al., 2023a; Hogwood et al., 2023b).

### 6.2 Precision medicine

High-throughput transcriptomics and artificial intelligence (AI) have been used to identify sepsis subphenotypes with different immune profiles and prognoses (Cano-Gamez et al., 2022). Whole-blood transcriptomic profiling has identified specific subclasses, such as pediatric Subclass 1, characterized by marked innate immune activation, adaptive immune suppression, and elevated biomarkers of endothelial injury (Yang J. O. et al., 2023). Separately, a whole-blood gene-expression reference map was used to derive a quantitative sepsis response signature (SRSq) score reflecting immune dysfunction and predicting clinical outcomes (Cano-Gamez et al., 2022).

Unsupervised analyses also identified clinical clusters. In the retrospective study by Guo et al. (2022), Cluster three had prolonged partial thromboplastin time, high SIC scores, and a higher proportion of patients receiving heparin. This describes treatment exposure within a prognostic cluster, not successful treatment attributable to heparin. Reinforcement-learning models have additionally explored heparin treatment policies using retrospective ICU data and computational value-function assessment (Liu J. et al., 2024). Such analyses may generate dosing hypotheses, but offline performance or similarity to clinician behavior does not demonstrate prospective efficacy, safety, or an individualized therapeutic range.

Integration of molecular and clinical phenotyping could inform enrollment and stratification in future trials. Neither the transcriptomic classes nor the computational treatment policies discussed here are validated criteria for selecting or dosing heparin for immunomodulation in sepsis.

### 6.3 Combination therapies

Combining heparin with NET-targeted interventions is an experimental hypothesis. Fuchs et al. (2010) showed that DNase or heparin could dismantle NET scaffolds and prevent thrombus formation in experimental systems; this does not establish clinical synergy between the two agents. DNase-based strategies and inhibition of NET formation, including PAD4-directed approaches, remain subjects of investigation (Zalghout and Martinod, 2025). Any proposed combination requires direct comparison with its individual components and evaluation of host defense, released NET-associated mediators, bleeding, and patient-centered outcomes. Benefit from combination treatment in patients with sepsis has not been established.

Clinical trials of other immunomodulators provide context for trial design but do not establish a benefit from combining them with heparin. Robey et al. (2024) reported a short-term mortality signal across heterogeneous cytokine-inhibitor trials, including an anti-TNF subgroup; the analysis does not justify a specific claim that IL-1 or IL-6 blockade combined with heparin improves survival. The PROVIDE trial investigated biomarker-guided immunotherapy with anakinra or interferon-gamma and reported an early organ-function signal, without establishing a 28-day survival benefit (Leventogiannis et al., 2022). Whether combining an immune-directed intervention with heparin yields additional efficacy or acceptable safety remains untested in the evidence reviewed here.

## 7 Conclusion

Synthesis of the current literature shows a change in how heparin is discussed in sepsis: it is considered not only as a prophylactic anticoagulant but also as a candidate multitarget immunomodulatory agent. Heparin has credible molecular actions beyond coagulation, and its chain length and sulfation permit antithrombin binding, histone neutralization, interference with HMGB1-LPS delivery, heparanase inhibition, and selectin antagonism. The evidence supporting these actions is, however, uneven and is dominated by biochemical, cellular, animal, human endotoxemia, biomarker, and non-sepsis studies. These mechanisms therefore establish biological plausibility, not a clinically proven immunomodulatory effect. The clinical picture must be stated with matching precision. Randomized evidence, including the most recent meta-analysis restricted to randomized trials, has not demonstrated a survival benefit of heparin in sepsis or sepsis-associated DIC (Totoki et al., 2024). Favorable associations in retrospective SIC cohorts and in data-derived high-risk classes justify prospective phenotype-enriched trials but do not identify established responders. On formulation, LMWH is generally preferred to UFH for routine ICU VTE prophylaxis where prophylaxis is indicated, but that recommendation cannot be extended to sepsis-specific anticoagulation or immunomodulation; no evidence supports choosing sodium over calcium heparin for sepsis outcomes, and lithium heparin is a laboratory reagent rather than a therapeutic product. There is accordingly no preferred preparation, salt, dose, or biomarker threshold for off-label immunomodulatory treatment of sepsis, and heparin should not be given for that purpose outside a clinical trial.

Nonetheless, the current literature is constrained by several important limitations. These include a small randomized evidence base of only a few hundred patients, historical reliance on fixed-dose regimens that do not account for individual patient trajectories, observational designs that remain vulnerable to confounding by contraindication and to time-related exposure bias, heterogeneous and often incompletely reported exposure definitions, sparse bleeding and heparin-induced thrombocytopenia reporting, and the diagnostic difficulty of distinguishing sepsis-induced coagulopathy from HIT.

Future studies should evaluate whether candidate biomarkers measure target engagement and whether specific heparin mimetics retain anti-inflammatory activity while reducing clinically important bleeding. Transcriptomic and computational phenotypes may inform prospective trial design, but require external and interventional validation before they can guide patient selection or dosing.
